# Seasonal Variations in Birth Patterns in Greece: A Comprehensive Analysis of National Data From 1956 to 2022

**DOI:** 10.7759/cureus.74235

**Published:** 2024-11-22

**Authors:** Nikolaos Vlachadis, Nikolaos Antonakopoulos, Dionysios N Vrachnis, Nikolaos Loukas, Alexandros Fotiou, Stamatoula Ouzouni, Konstantinos Louis, Christos Parthenis, Marianna Theodora, Nikolaos Vrachnis

**Affiliations:** 1 Department of Obstetrics and Gynecology, General Hospital of Messinia, Kalamata, GRC; 2 Department of Obstetrics and Gynecology, School of Health Sciences, University of Patras, Patras, GRC; 3 Third Department of Obstetrics and Gynecology, National and Kapodistrian University of Athens, Attiko Hospital, Athens, GRC; 4 Department of Obstetrics and Gynecology, Tzaneio Hospital, Piraeus, GRC; 5 Department of Maternal-Fetal Medicine, Elena Venizelou Hospital, Athens, GRC; 6 First Department of Obstetrics and Gynecology, National and Kapodistrian University of Athens, Alexandra Hospital, Athens, GRC

**Keywords:** births, fertility, greece, seasonality, seasonal variation, time trends

## Abstract

Introduction: The seasonality of human births has been studied globally for over two centuries, revealing diverse patterns across populations shaped by intricate interactions involving both biological and socio-cultural factors. This study offers a thorough examination of national birth data in Greece spanning from 1956 to 2022, aiming to elucidate long-term trends and changes in seasonal birth patterns.

Materials and methods: Data on live births in Greece were categorized by month based on national registries, and the analysis of birth seasonality was conducted annually. The Edwards method was utilized to evaluate birth seasonality by calculating the peak angle (PA), which identifies the center of the seasonal birth distribution. Additionally, the peak-to-low ratio (PLR) was computed for each year to serve as an indicator of the amplitude of birth seasonality. To investigate trends and temporal changes, joinpoint regression analysis was employed, specifically focusing on the annual percentage change (APC) within defined segments, along with a 95% confidence interval (95% CI).

Results: A total of 8,144,465 live births were recorded between 1956 and 2022. Birth seasonality was statistically significant for all examined years. The results revealed a progressive shift in the concentration of births to the second half of the year, transitioning from a peak in January and February to two significant major peaks in July and September. Additionally, in the last two decades, the lowest birth rates were observed in spring, particularly in April, as well as in December. The PA shifted significantly over time, moving from mid-January in 1956 to late August in 2022, with an APC of 39.6 (95% CI: 17.4 to 99.7) during 1956-1960 and 7.5 (95% CI: 2.2 to 11.3) during 1960-1975. Between 1975 and 2022, the trend continued at a lower stable rate (APC = 1.0, 95% CI: 0.2 to 1.5). The PLR, after a brief upward trend during 1956-1960 (APC = 6.0, 95% CI: 3.1 to 11.3), exhibited a statistically significant decline in seasonal amplitude from 1960 to 1992 (1960-1964: APC = -5.5, 95% CI: -8.6 to -2.4, 1964-1992: APC = -0.4, 95% CI: -0.8 to -0.2), and relatively stabilized during the last thirty years (1992-2022).

Conclusions: This study highlights significant changes in birth seasonality in Greece, marked by a shift in the distribution of births toward the second half of the year and a transition from peaks in births during January and February to two major surges in July and September. These trends reflect evolving reproductive behaviors in the Greek population and hold important implications for reproductive and perinatal healthcare in the country.

## Introduction

The phenomenon of seasonality in human births has been researched and recorded for two centuries across various populations. Nevertheless, this rhythmic pattern is not consistent, as significant differences and temporal variations in its characteristics have been observed in different countries and geographical regions. While the seasonal effects on reproduction have been thoroughly studied in mammals, there is limited information regarding the impact of seasonal variation and its underlying mechanisms on human reproduction [1,2].

The seasonal patterns of births correspond closely to the temporal patterns of human conceptions. While the exact causes of these seasonal variations remain largely unknown, they are likely influenced by a combination of biological, cultural, and socio-economic factors. Research indicates that several human reproductive factors, including male sperm quality, ovulation rates, and the frequency of sexual intercourse, may exhibit annual variability. Additionally, environmental factors like temperature, daylight duration, and exposure to light influence normal reproductive functions [3,4]. Furthermore, social and cultural elements, such as religious holidays, vacation periods, and the seasonality of marriages, can affect the frequency of conception at various times of the year within specific populations [5-7].

The evolutionary approach to human seasonal reproductive diversity suggests that fecundity peaks in summer, resulting in increased natality in spring. This pattern allows for approximately six months of favorable environmental conditions for newborns, a trend that has been observed in northern countries [1,6]. The two primary annual patterns of births are the "European" model, which features a significant peak in births during spring (specifically in April) and a smaller peak in early autumn, and the "American" model, which, in contrast, shows a notable decline in spring, with its highest birth rates occurring in August and September [8].

Furthermore, the study of seasonal variation in births is also important because the timing of an individual's birth appears to correlate with significant epidemiological parameters and the risk of developing various chronic diseases throughout their lifetime. These diseases include cardiovascular conditions and psychiatric and neurological disorders. Additionally, longevity has also been found to correlate with the season of birth [9,10].

Greece has experienced a significant decline in birth rates over recent decades, alongside a notable increase in births among older women and a high prevalence of medically assisted reproduction [11]. This study aims to elucidate the seasonal variations in birth patterns in Greece through a comprehensive analysis of national data spanning from 1956 to 2022.

## Materials and methods

Data on live births in Greece from 1956 to 2022, categorized by month of delivery and sourced from birth registrations provided by the Hellenic Statistical Authority, were analyzed. An Edwards analysis of birth seasonality was conducted by calculating the peak angle (PA) of the distribution for each year, which typically indicates the timing of the peak value of the seasonal component for each year. The PA is expressed in degrees, with 0 degrees representing the beginning of the year.

To account for the varying lengths of months, we calculated the average daily number of live births for each month, as well as the average daily number of live births for each year. Subsequently, the seasonal index (SI) for each month in each year was determined by dividing the observed daily number of births by the average daily number of births for the year and then multiplying the result by 100. A value of 100 signifies the average annual number of births, representing the expected number of births in the absence of monthly seasonal fluctuations. For instance, an SI value of 110 indicates that, in a specific month, the observed number of births was 10% higher than the annual average or expected figure. The peak-to-low ratio (PLR) was calculated for each year as the quotient of the highest monthly mean number of births observed during that year to the lowest monthly mean number of births within the same year. In the context of seasonality analysis, the PLR serves as a metric to evaluate the relative strength of seasonal variations within a dataset. A higher PLR indicates greater amplitude and more pronounced seasonal effects, while a lower PLR suggests less variability and weaker seasonal patterns.

Birth data analysis was conducted using Microsoft Excel (Microsoft Corp., Redmond, WA), while Edwards analysis was performed with Episheet software (RTI Health Solutions, Research Triangle Park, NC). Trends in PA, annual peak and low SI, and PLR were assessed using the Joinpoint Regression Program, version 5.2.0 (National Cancer Institute, Bethesda, MD). This analysis facilitates the identification of joinpoints, which represent the years in which statistically significant changes in trend occur. The annual percentage change (APC) was calculated for each segment between two joinpoints, allowing for a maximum of seven segments with a 95% confidence interval (95% CI). A p-value of less than 0.05 was considered statistically significant.

## Results

The total number of live births from 1956 to 2022 was 8,144,465. The highest average daily number of births occurred in January 1959 with 566.8 births, while the lowest was recorded in April 2022 with 185.6 births.

**Figure 1:**
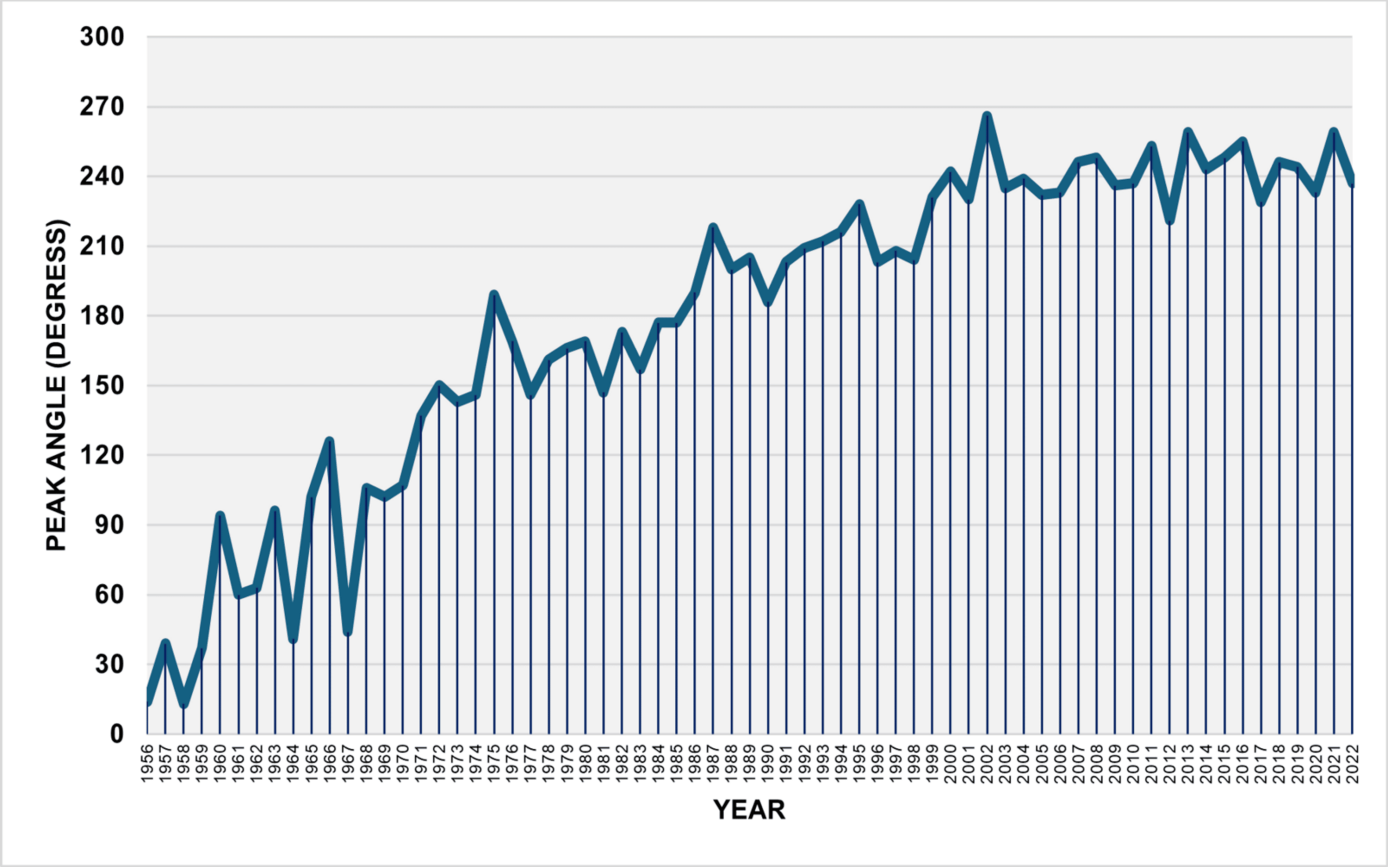
Peak angle of the seasonal birth distribution in Greece, 1956–2022 The peak angle indicates the timing of the peak in births for each year. It was calculated using Edwards' analysis and is expressed in degrees, with 0° corresponding to the first day of the year.

The PA was statistically significant (p < 0.001) for all years and increased from 14 degrees in 1956 to 237 degrees in 2022, reflecting the movement from the beginning of the year (15 January) to the end of summer (29 August). The minimum value recorded was 13 degrees in 1958, which corresponds to mid-January (14 January), while the maximum value reached 266 degrees in 2002, corresponding to late September (27 September). The PA ranged from January to February in the 1950s, exhibiting significant fluctuations during the 1960s, moving from February to May. In the 1970s, the PA was observed from April to June, except for 1975. Since the mid-1980s, the PA of births has shifted completely to the second half of the year. Over the last 24 years (1999-2022), the PA has typically been observed in the latter half of August and September, except for 2012, when it was recorded slightly earlier than mid-August (Figure 1).

**Figure 2:**
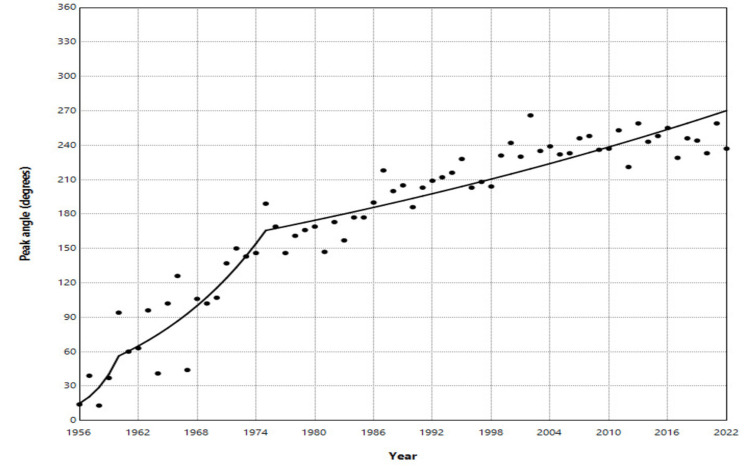
Trends in the peak angle of the seasonal birth distribution in Greece, 1956–2022 The peak angle indicates the timing of the annual peak in births. It was calculated using Edwards' analysis and is expressed in degrees, with 0° corresponding to the first day of the year. Trends were assessed using joinpoint regression analysis. Statistical values are presented in Table 1.

There was a notable increasing trend in PA, particularly steep during the initial four years from 1956 to 1960, with an APC of 39.6 (95% CI: 17.4 to 99.7, p < 0.001). This was followed by a rapid rise over the subsequent 15 years, from 1960 to 1975, with an APC of 7.5 (95% CI: 2.2 to 11.3, p < 0.006). In the last five decades, from 1975 to 2022, the shift in the seasonal birth apex towards the second half of the year persisted, with an APC of 1.0 (95% CI: 0.2 to 1.5, p = 0.044) (Figure 2, Table 1).

**Table 1:** Trends in the peak angle of the seasonal birth distribution in Greece, 1956–2022 The peak angle indicates the timing of the annual peak in births. Trends were assessed using joinpoint regression analysis. A p-value < 0.05 was considered statistically significant.

Segment	Annual percentage change	95% confidence interval	P-value
1956-1960	39.6	17.4 to 99.7	0.001
1960-1975	7.5	2.2 to 11.3	0.006
1975-2022	1.0	0.2 to 1.5	0.044

**Figure 3:**
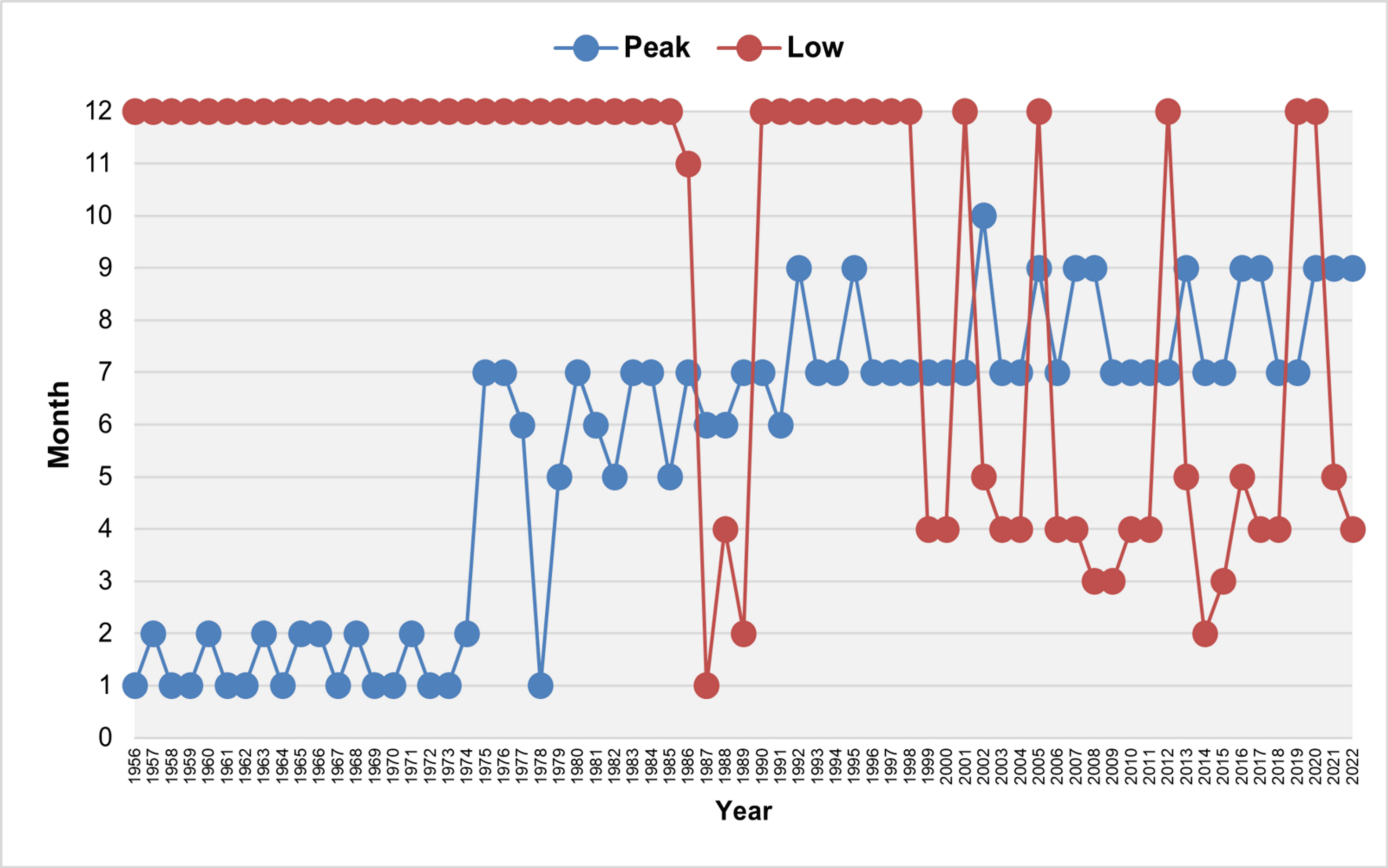
Peak and low birth months in Greece, 1956–2022 The peak birth month for each year is the month with the highest number of births, while the low birth month is the month with the fewest births, after adjusting for the varying lengths of months. Months are listed in numerical order (1 for January to 12 for December).

Figure 3 illustrates the month with the highest number of births and the month with the lowest number of births for each year.

**Figure 4:**
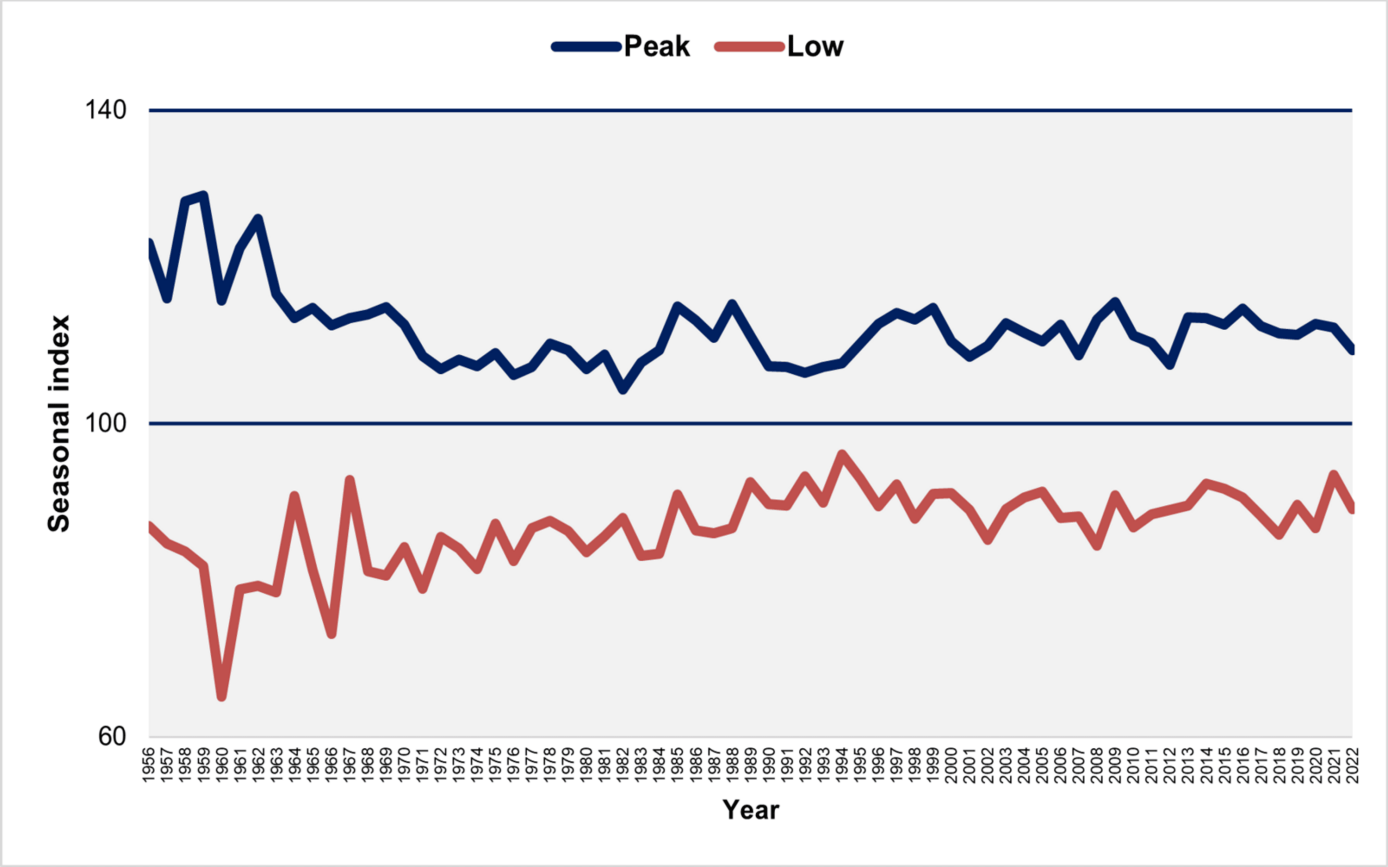
Seasonal index of peak and low birth months in Greece, 1956–2022 The seasonal index for each month of each year was calculated by dividing the observed daily number of births by the average daily number of births for the year, then multiplying the result by 100 to account for varying month lengths. A value of 100 indicates the average daily number of births, representing the expected number in the absence of monthly seasonal fluctuations.

The peak birth month was January from 1956 to 1974. However, from 1975 to 2022, the peak months shifted to July and September, with exceptions in only 10 years: January (1978), May (1979, 1982, 1985), June (1977, 1981, 1987, 1988, 1991), and October (2002). Over the last 34 years, most births occurred in July or September, with the exceptions of 1991 and 2002. In the last three years (2020-2022), September emerged as the leading month, with a SI ranging from 112.8 in 2020 to 109.3 in 2022 (Figures 3, 4).

**Figure 5:**
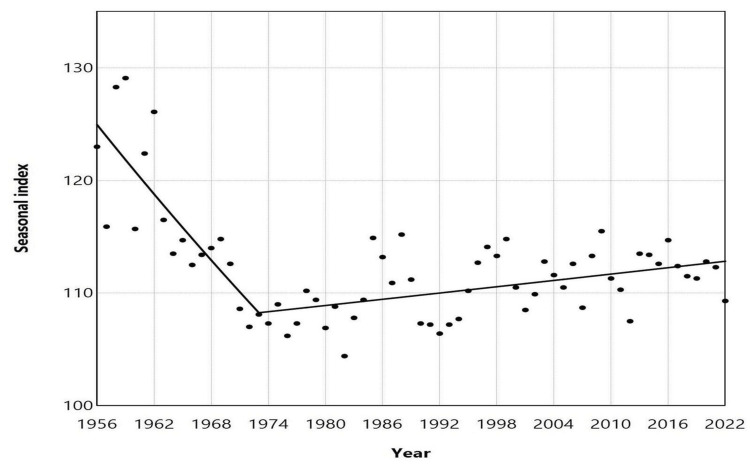
Trends in the seasonal index of peak birth months in Greece, 1956–2022 The seasonal index for each month of each year was calculated by dividing the observed daily number of births by the average daily number of births for the year, then multiplying the result by 100 to account for varying month lengths. Trends were assessed using joinpoint regression analysis. Statistical values are presented in Table 2.

The SI of the annual peak natality month ranged from a maximum of 129.1 in January 1959 to a minimum of 104.4 in May 1982, with a median value of 111.5 and an interquartile range (IQR) of 108.7 to 114.0. The seasonal index of the annual peak natality month ranged from a maximum of 129.1 in January 1959 to a minimum of 104.4 in May 1982, with a median value of 111.5 and an IQR of 108.7 to 114.0. This indicates that the median peak monthly natality was 11.5% higher than the annual average (Figure 4). Furthermore, a downward trend of maximum SI was observed from 1956 to 1973 (APC = -0.8, 95% CI: -1.2 to -0.6, p < 0.001), followed by a gradual upward trend from 1973 to 2022 (APC = 0.1, 95% CI: 0.0 to 0.1, p = 0.002) (Figure 5, Table 2).

**Table 2:** Trends in the seasonal index of peak birth months in Greece, 1956–2022 The seasonal index for each month of each year was calculated by dividing the observed daily number of births by the average daily number of births for the year, then multiplying the result by 100 to account for varying month lengths. Trends were assessed using joinpoint regression analysis. A p-value < 0.05 was considered statistically significant.

Segment	Annual percentage change	95% confidence interval	P-value
1956-1973	-0.8	-1.2 to -0.6	< 0.001
1973-2022	0.1	0.0 to 0.1	0.002

From 1956 to 1998, December consistently recorded the lowest birth rate, except for the years 1986 to 1989, during which the lowest months were November (1986), January (1987), April (1988), and February (1989). Over the past 24 years, the months with the lowest birth rates have generally been from January to May. However, December has also recorded the lowest birth rates in five specific years: 2001, 2005, 2012, 2019, and 2020 (Figure 4).

**Figure 6:**
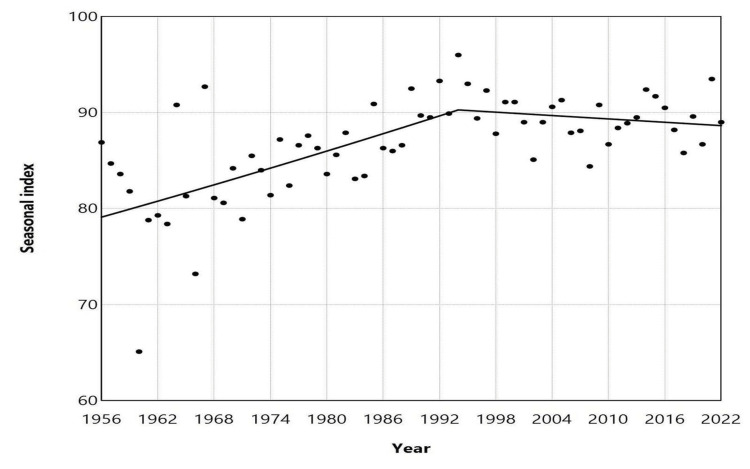
Trends in seasonal index of low birth months in Greece, 1956-2022 The seasonal index for each month of each year was calculated by dividing the observed daily number of births by the average daily number of births for the year, then multiplying the result by 100 to account for varying month lengths. Trends were assessed using joinpoint regression analysis. Statistical values are presented in Table 3.

The SI for the month with the fewest annual births varied from a low of 65.1 in December 1960 to a high of 96.0 in December 1994, with a median value of 87.6 (IQR: 84.0 to 90.5). This suggests that the median low monthly birth rate was 12.4% lower than the annual average (Figure 4). The SI demonstrated an upward trend from 1956 to 1994, with an APC of 0.3 (95% CI: 0.2 to 0.5, p < 0.001). However, for the more recent period from 1994 to 2022, the trend stabilized (APC = -0.1, 95% CI: -0.3 to 0.2, p = 0.557) (Figure 6, Table 3).

**Table 3:** Trends in the seasonal index of low birth months in Greece, 1956–2022 The seasonal index for each month of each year was calculated by dividing the observed daily number of births by the average daily number of births for the year, then multiplying the result by 100 to account for varying month lengths. Trends were assessed using joinpoint regression analysis. A p-value < 0.05 was considered statistically significant.

Segment	Annual percentage change	95% confidence interval	P-value
1956-1994	0.3	0.2 to 0.5	< 0.001
1994-2022	-0.1	-0.3 to 0.2	0.557

**Figure 7:**
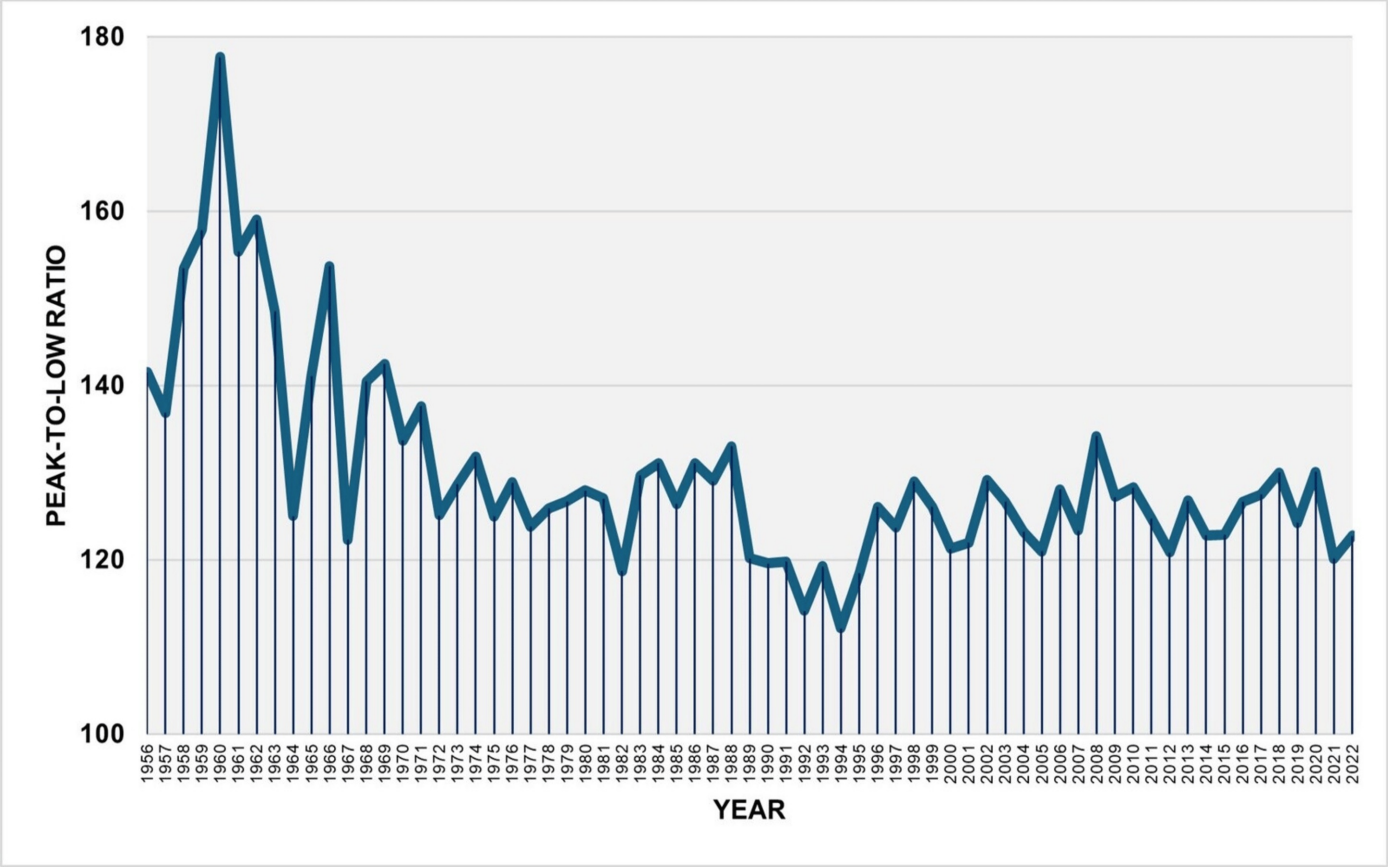
Peak-to-low ratio of monthly births in Greece, 1956–2022 The peak-to-low ratio is a measure of the relative strength of seasonal variation for each year, calculated as the quotient of the highest monthly mean number of births to the lowest monthly mean number of births within the same year.

**Figure 8:**
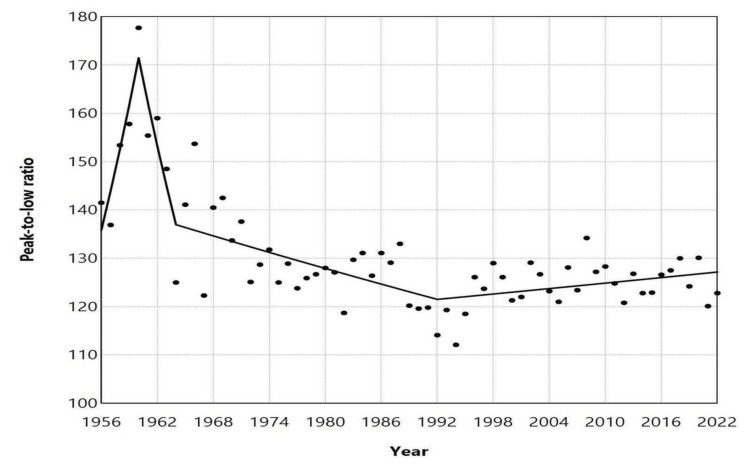
Trends in the peak-to-low ratio of monthly births in Greece, 1956–2022 The peak-to-low ratio is a measure of the relative strength of seasonal variation for each year, calculated as the quotient of the highest monthly mean number of births to the lowest monthly mean number of births within the same year. Trends were assessed using joinpoint regression analysis. Statistical values are presented in Table 4.

The PLR varied from a maximum of 177.7 in 1960 to a minimum of 112.1 in 1994, with a median of 126.8 (IQR of 122.9 to 131.8). There was an upward trend from 1956 to 1960, with an APC of 6.0 (95% CI: 3.1 to 11.3, p = 0.002). This was followed by a downward trend from 1960 to 1964 with an APC of -5.5 (95% CI: -8.6 to -2.4, p = 0.002) and from 1964 to 1992 with an APC of -0.4 (95% CI: -0.8 to -0.2, p = 0.006). In the more recent 30-year period from 1992 to 2022, the trend showed a slight increase, although it did not reach statistical significance (APC = 0.2, 95% CI: -0.0 to 0.8, p = 0.084). Since 1996, the PLR has fluctuated between 120.1 and 134.2 (Figures 7, 8, Table 4). 

**Table 4:** Trends in the peak-to-low ratio of monthly births in Greece, 1956–2022 The peak-to-low ratio measures the relative strength of seasonal variation for each year, calculated as the quotient of the highest monthly mean number of births divided by the lowest monthly mean number of births within the same year. Trends were assessed using joinpoint regression analysis. A p-value < 0.05 was considered statistically significant.

Segment	Annual percentage change	95% confidence interval	P-value
1956-1960	6.0	3.1 to 11.3	0.002
1960-1964	-5.5	-8.6 to -2.4	0.002
1964-1992	-0.4	-0.8 to -0.2	0.006
1992-2022	0.2	-0.0 to 0.8	0.084

**Figure 9:**
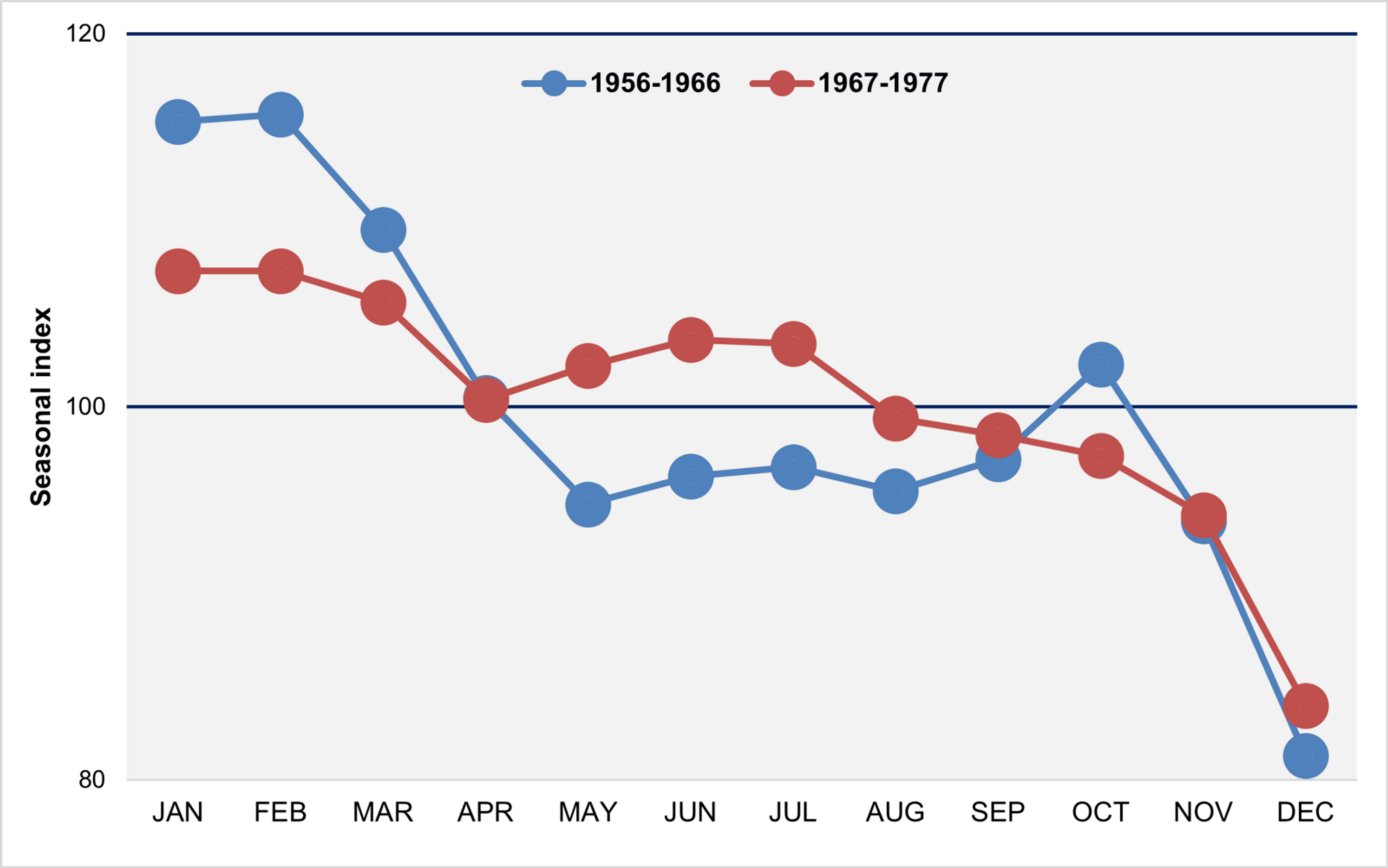
Median monthly seasonal index of births in Greece, 1956–1966 and 1967–1977 The seasonal index for each month of each year was calculated by dividing the observed daily number of births by the average daily number of births for the year, then multiplying the result by 100 to account for varying month lengths. A value of 100 represents the average daily number of births, reflecting the expected number in the absence of monthly seasonal fluctuations.

Between 1956 and 1966, the monthly distribution of births appears bimodal, characterized by a peak in January and February, a secondary peak in October, and two troughs: a moderate one in May and a pronounced low in December. The data for 1967-1977 also indicated a less pronounced peak in January and February, followed by a secondary peak during the summer months of June and July. The last five months of the year consistently fell below the average, with December showing the most significant decline (Figure 9).

**Figure 10:**
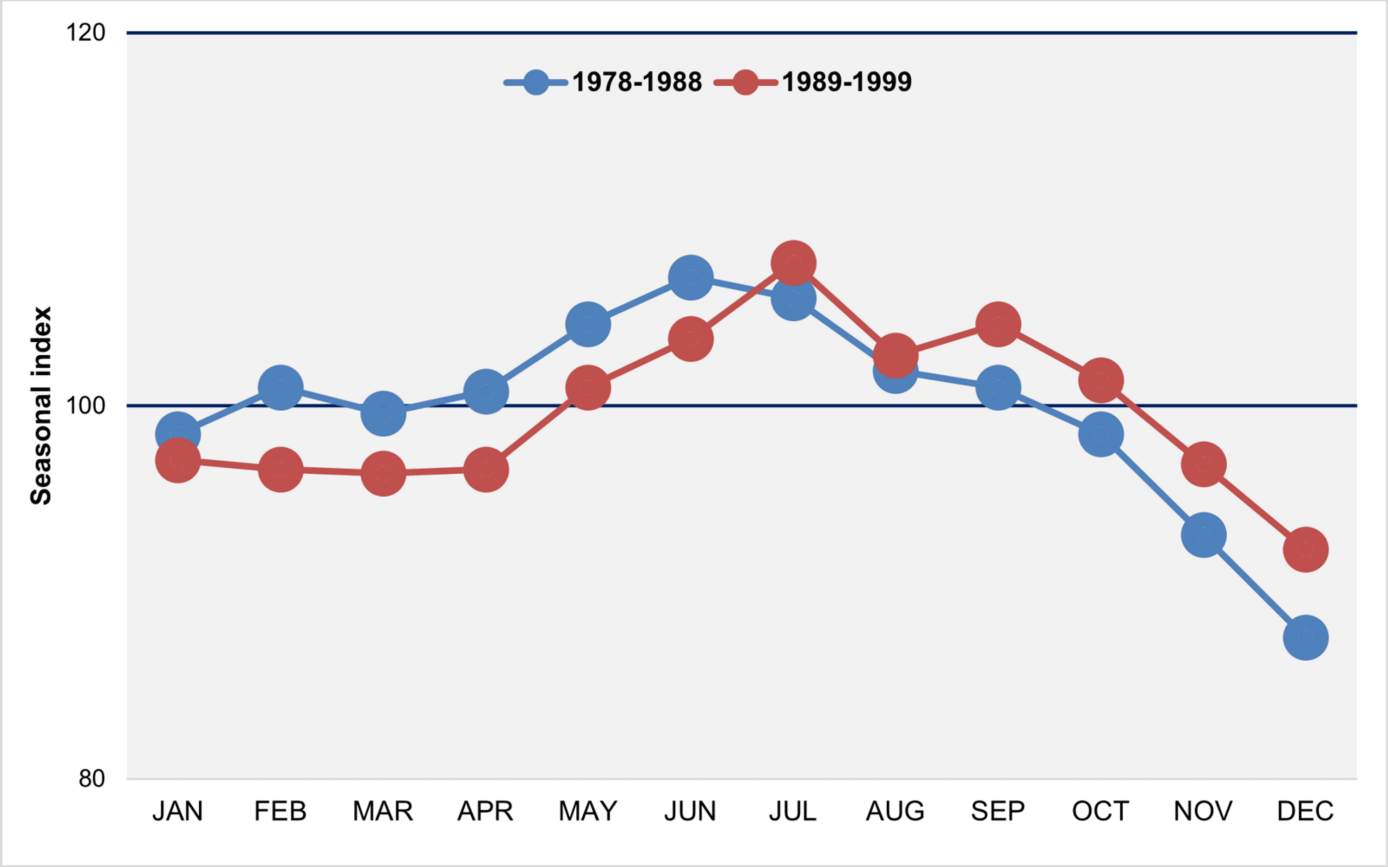
Median monthly seasonal index of births in Greece, 1956–1966 and 1967–1977 The seasonal index for each month of each year was calculated by dividing the observed daily number of births by the average daily number of births for the year, then multiplying the result by 100 to account for varying month lengths. A value of 100 represents the average daily number of births, reflecting the expected number in the absence of monthly seasonal fluctuations.

In the decade spanning from 1978 to 1988, the distribution displayed a unimodal pattern, characterized by a peak in June and a notable low in January, accompanied by a pronounced trough in December. During the period from 1989 to 1999, the distribution remained unimodal, with a peak occurring in July and a significant trough also observed in December (Figure 10).

**Figure 11:**
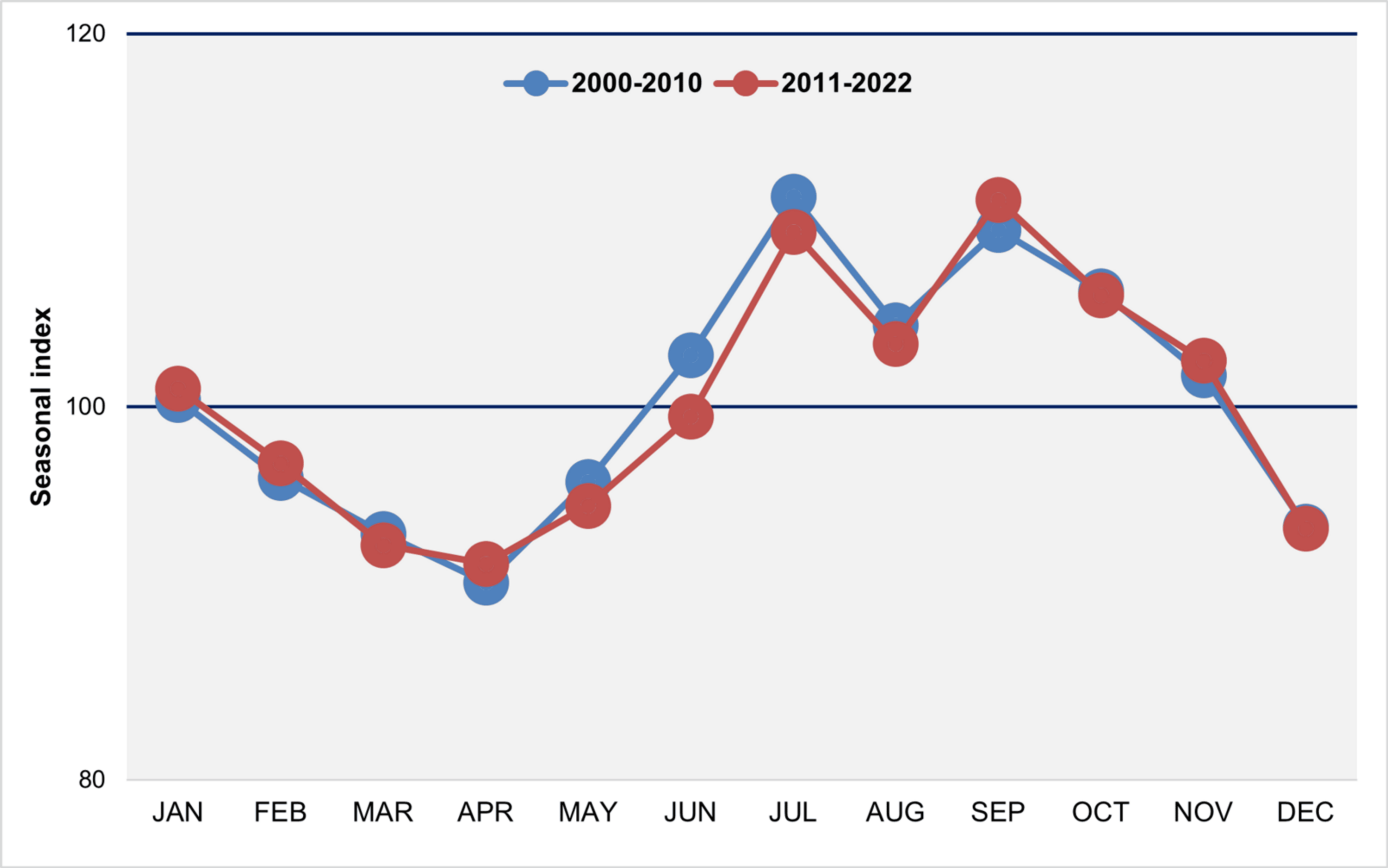
Median monthly seasonal index of births in Greece, 2000-2010 and 2011-2022 The seasonal index for each month of each year was calculated by dividing the observed daily number of births by the average daily number of births for the year, then multiplying the result by 100 to account for varying month lengths. A value of 100 represents the average daily number of births, reflecting the expected number in the absence of monthly seasonal fluctuations.

In the periods 2000-2010 and 2011-2022, the distributions exhibit similarities, displaying a characteristic bimodal configuration with peaks typically occurring in July and September and a dip in August. Additionally, there are two troughs observed in April and December, respectively (Figure 11).

## Discussion

This study examined natality patterns within the Greek population by analyzing national birth data over nearly seven decades. The results revealed a progressive shift in the concentration of births from the first half of the year to the second half, with a notable transition from a peak in January and February to two significant major peaks in July and September. Moreover, in the last two decades, the lowest points in the birth distribution were observed in spring, particularly in April, as well as in December.

The analysis revealed a statistically significant upward trend in PA from 1956 to 2022, particularly steep until 1960, with an APC of 39.6%. Following this, the rate of increase remained rapid at an average of 7.5% per year from 1960 to 1975, before slowing to an average annual rate of 1% from 1975 to 2022. The PA indicates the center of the dominant seasonal effect, which has clearly transitioned into the second half of the year since the mid-1980s. Notably, since 2000, fluctuations in the PA have predominantly occurred during late August and throughout September.

Additionally, the annual PLR was calculated to assess the intensity of seasonal variations in births. The PLR experienced significant increases, reaching a historical high of 177.7 in 1960, followed by a sharp decline until 1964, with an APC of 5.5%. A steady contraction in birth seasonality continued at an annual rate of 0.4%, culminating in a historical low in 1994 when the month of highest births and the month of lowest births differed by only 12.1%. Over the past three decades, the total amplitude of birth seasonality has exhibited relative stability, fluctuating between approximately 20% and 34%.

Two principal patterns of human birth seasonality have been identified. The 'European' pattern is characterized by a higher birth rate in the spring, corresponding to an increase in conceptions during the summer months. Conversely, the 'American' pattern exhibits a decline in births during spring, linked to a decrease in summer conceptions. A shared feature of both patterns is a peak in births in September, which aligns with an increase in conceptions around the Christmas holiday [3,8]. In Greece, the pattern of monthly birth seasonality has undergone significant changes over the decades. During the period from 1956 to 1966, there was a pronounced increase in birth rates during the first two months of the year, with a secondary, smaller peak in October. This pattern closely resembled the 'European' model, though with the peak occurring about two months earlier. Over time, there has been a gradual shift towards the 'American' pattern, notably prominent in the last two decades, characterized by a sharp decline in spring births and a clear predominance of births in July and September. A similar shift has been observed in other European countries, such as the Netherlands, attributed to concurrent reproductive changes in the population, including declining fertility rates and lower parity [2].

The analysis of monthly birth seasonality in Greece revealed a distinct shift in the distribution of births from the first to the second half of the year. While January remained the peak birth month until 1974, the last three decades have been dominated by July and September. In the most recent years (2020-2022), September has consistently recorded the highest number of births, with a peak-to-mean amplitude ranging from approximately 9% to 12%. Although December had been the month with the fewest births for over forty years, the past two decades have seen the lowest birth rates shift to the period between January and May, although December has remained a secondary low point. Since 2000, a characteristic feature of the monthly birth pattern in Greece has been a decline in births during August, positioned between the two peaks in July and September. This trend is likely attributable to August being the primary summer holiday month.

A time series analysis of national data from Spain showed a similar pattern of birth seasonality in the 1950s and 1960s, with a peak in March and a smaller rise in October, gradually diminishing and disappearing by the 1990s [3]. The reduction in seasonality intensity was similarly evident in the Greek data up to approximately the mid-1990s, likely reflecting gradual urbanization and a shift away from traditional perspectives. However, over the past three decades, the degree of seasonal variation in birth rates has reached a relative stabilization, exhibiting a slight (statistically non-significant) increasing trend. Since 1996, the amplitude of birth seasonality in Greece has remained substantial, with fluctuations between approximately 20% and 34%. This shift may be linked to increased migration with diverse cultural characteristics, a significant decline in birth rates within the Greek population, and a considerable rise in maternal age, coinciding with an increase in the use of assisted reproductive technology [2,11,12]. Notably, the number of in vitro fertilization treatments exhibits significant seasonal variations [13].

Two studies examined the seasonality of births in Greece prior to 1980. The first study, aligning with the findings of the present analysis, identified a birth pattern for the period 1956-1974 characterized by peaks in January and February, primarily attributed to cultural and religious factors, while a predominance of births occurred from May to July during 1974-1979 [14]. The second study found a gradual shift in peak births from January to June and a decrease in seasonality amplitude, revealing a strong correlation between recent seasonal variations in birth patterns and the seasonality of marriages [7]. Both studies emphasized December as the month with the lowest birth rate in Greece, likely reflecting reduced conceptions during the Great Lent preceding Orthodox Easter, with the rise in births in January associated with increased coital rates following the Easter holiday. Both studies emphasized the consistent position of December as the month with the lowest birth rate in Greece. The decline in December births is likely closely related to the reduction in conceptions during the Great Lent period that precedes Orthodox Easter, whereas the rise in births in January is associated with an increase in the coital rate following the Easter holiday [7,14].

A recent study examining conceptions across various population subgroups in Greece over five decades identified notable variations in seasonality patterns between mountainous and lowland populations, although these effects diminished over time [15]. Another study focusing on birth seasonality in Eastern and Central European countries from 1996 to 2012 found two distinct peaks: one in July and another in September [16]. Birth seasonality appears to vary by latitude; data from the United States indicate that northern states experience peaks in spring and summer, while southern states see peaks in autumn [17].

While the seasonality of births is well documented, the underlying causes of these seasonal variations remain poorly understood. Human birth seasonality is likely influenced by a complex interplay of biological, environmental, socioeconomic, and cultural factors [5]. Environmental conditions, including temperature, humidity, and daylight, can affect female fertility and the likelihood of conception. Seasonal fluctuations in hormone levels, such as gonadotropins and melatonin, may also impact reproductive processes. Evidence suggests a seasonal pattern in ovulation, oocyte maturation, endometrial receptivity, and frequency of intercourse. Furthermore, semen concentrations and counts generally decline in summer, possibly due to heat and shorter day lengths [1,18,19]. The seasonality of births may reflect evolutionary adaptations in early societies, optimizing offspring survival by aligning births with periods of abundant resources, favorable climates, and minimum exposure to infections [1,20]. In contrast, modern social and cultural factors, such as holidays and traditions, significantly influence the timing of births. Additionally, important demographic factors, including parity, maternal age, educational attainment, and economic status, play crucial roles in shaping the extent of birth seasonality [5,19,21].

The study of birth seasonality should be complemented by an analysis of the seasonality of major pregnancy complications. Significant seasonal variations in the incidence of preterm births have been documented [22,23]. Research on the Greek population has also noted seasonality in preterm births, identifying peaks in summer and winter [24]. These findings may significantly impact seasonal birth patterns in Greece, where the incidence of preterm births is notably high [25,26]. Additionally, an increased frequency of early spontaneous miscarriages has been reported in late summer [27].

Establishing a seasonal pattern of births has important implications for healthcare delivery, societal understanding, and policy-making. Investigating birth seasonality sheds light on human reproduction biology and the effects of environmental conditions on fetal development and pregnancy outcomes. This knowledge is vital for devising strategies to enhance perinatal health through targeted interventions across different seasons. Furthermore, examining the social and cultural factors influencing birth timing provides insights into population-specific birth patterns [28]. Economically, recognizing birth seasonality facilitates effective planning of healthcare resources, as variations in birth rates throughout the year affect medical systems. Finally, an intriguing aspect of researching seasonal variations in births is the epidemiological evidence linking birth month to lifetime risks of various chronic diseases in both childhood and adulthood, including psychiatric, neurological, immunological, and cardiovascular conditions [9]. Additionally, research indicates that the season of birth, combined with climatic factors, may impact female fecundity later in life [29]. These effects could be driven by early developmental changes and DNA methylation influenced by the season of birth [9,30].

A key limitation of the current study is that the analysis of birth data was restricted to the national level. Additional research investigating seasonality in relation to factors such as geographical region, birth order, and socioeconomic status could yield greater insights into the impact of these parameters on long-term trends and the extent of birth seasonality within the Greek population.

## Conclusions

This comprehensive analysis of seasonal variations in birth patterns in Greece from 1956 to 2022 highlights significant shifts in natality trends over the decades. The findings indicate a notable shift in the concentration of births from the first half of the year to the second half, marked by a transition from earlier peaks in January and February to peaks in July and September, alongside minimums occurring in spring and December. These insights enhance our understanding of reproductive behaviors within the Greek population and have important implications for policy planning and resource allocation in perinatal healthcare.
